# Impact of Cancer-Related Virtual Visits on Travel Distance, Travel Time, and Carbon Dioxide (CO_2_) Emissions during the COVID-19 Pandemic in Manitoba, Canada

**DOI:** 10.3390/curroncol30070446

**Published:** 2023-06-21

**Authors:** Pascal Lambert, Grace Musto, Maclean Thiessen, Piotr Czaykowski, Kathleen Decker

**Affiliations:** 1 CancerCare Manitoba Research Institute, CancerCare Manitoba, 675 McDermot Avenue, Winnipeg, MB R3E 0V9, Canada; plambert@cancercare.mb.ca; 2 Department of Epidemiology and Cancer Registry, CancerCare Manitoba, 675 McDermot Avenue, Winnipeg, MB R3E 0V9, Canada; gmusto@cancercare.mb.ca; 3 Department of Medical Oncology and Hematology, CancerCare Manitoba, 675 McDermot Avenue, Winnipeg, MB R3E 0V9, Canada; maclean.thiessen@umanitoba.ca (M.T.); pczaykowski@cancercare.mb.ca (P.C.); 4 Department of Internal Medicine, Rady Faculty of Health Sciences, Max Rady College of Medicine, University of Manitoba, 820 Sherbrook Street, Winnipeg, MB R3E 0V9, Canada; 5 Department of Community Health Sciences, Rady Faculty of Health Sciences, Max Rady College of Medicine, University of Manitoba, 750 Bannatyne Avenue, Winnipeg, MB R3E 0V9, Canada

**Keywords:** cancer, virtual care, greenhouse gas emissions, COVID-19

## Abstract

CancerCare Manitoba (CCMB) introduced virtual visits at the beginning of the COVID-19 pandemic to replace many in-person visits. This study examines the impact of virtual visits for cancer care on travel distance, travel time, and carbon dioxide (CO_2_) emissions. We included all visits to CCMB for invasive and in situ cancers from 1 April 2020 to 31 December 2022. Data were extracted from CCMB’s electronic health record. The percentage of visits done virtually by month was reported by age, gender, cancer diagnosis, and regional health authority of residence. Postal codes for patients’ residences and clinic locations were converted into latitude and longitude values. Travel distance, travel time, and CO_2_ emissions associated with travel were estimated. The percentage of virtual visits was highest during the months when COVID-19 restrictions were present in Manitoba and represent more than 50% of such monthly visits. Virtual visits increased with age, were highest among men with urogenital cancer, and were lowest among northern Manitoba residents. The median travel time per visit ranged from 30 min in Winnipeg to 15 h in the Northern Region. The estimated travel distance saved varied from 420,000 to 750,000 km per month. Estimated travel time saved varied from 5500 to 9600 h per month. Estimated CO_2_ emissions prevented varied from 87 to 155 metric tons per month. Virtual care is an important tool for better supporting those living with cancer by substantially decreasing travel distance and time. Virtual care also contributes to reducing greenhouse gas emissions.

## 1. Introduction

Due to the increased vulnerability of individuals diagnosed with cancer to infections with COVID-19 [1,2,3,4], CancerCare Manitoba (CCMB), the provincial agency responsible for cancer treatment, introduced virtual visits midway through March 2020. For some appointments, instead of an in-person visit, individuals could remain in their homes and interact with CCMB health care providers through telephone and videoconferencing (Microsoft Teams application) [5]. This was in addition to the telemedicine already used in Manitoba (Manitoba Telehealth) prior to the pandemic where individuals could travel to a health care facility and have a videoconference with a health care provider at another facility [6]. Research has indicated that telemedicine visits provide direct financial benefits to patients through reduced travel and production losses (e.g., absence from employment), as well as environmental benefits through reduced carbon dioxide (CO_2_) emissions [7,8,9,10,11,12]. Because virtual visits for cancer care in Manitoba began in 2020, little research examining their impact has been done. The objectives of this study were to describe the patterns of visit types (in-person versus virtual) over time during the pandemic at CCMB, as well as the impact of virtual visits on hypothetical travel distance, travel time, and CO_2_ emissions generated by travel.

## 2. Materials and Methods

### 2.1. Data Sources

All visits for invasive and in situ cancers including benign brain and central nervous system tumours from 1 April 2020 to 31 December 2022 for Manitoba residents were extracted from CCMB electronic health records (ARIA MO and RO). The month of March 2020 was excluded because COVID-19-related restrictions were introduced during the middle of the month. Visits were either in-person or virtual (i.e., phone or Teams meeting from the patient’s residence). All Manitoba Telehealth visits from 1 January 2019 to 31 December 2022 were also extracted. Manitoba Telehealth provides videoconferencing through many facilities across Manitoba for health care services, continuing education, meetings, and family visits [6]. With this service, cancer patients can access a Manitoba Telehealth location to interact with CCMB health care providers located at another clinic. For the purposes of this study, Manitoba Telehealth visits were combined with in-person visits because of the requirement of travel to a health care facility. In addition, Manitoba Telehealth visits were available prior to the pandemic and the study objective is to evaluate the impact of implementing virtual visits. The following variables were extracted: age at visit, gender, last cancer diagnosed prior to the visit, regional health authority (RHA) of residence at time of visit, date and time of visit, postal code of the patient’s residence, and postal code of the clinic where health care provider was located. Regional health authority of residence was based on Manitoba’s five RHAs which include the Winnipeg Regional Health Authority, Interlake–Eastern, Southern Health–Santé-Sud, Prairie Mountain Health, and Northern Health Region (Figure S1 in the supplement). Manitoba Health includes the town of Churchill in the Winnipeg Regional Health Authority because residents in Churchill travel to the Winnipeg Regional Health Authority to receive health care. However, to reflect where an individual lives and not where they receive care, we have included Churchill as part of the Northern Health Region. 

Postal codes were converted to latitude and longitude values using Postal Code Conversion File Plus (PCCF+) [13]. Geographical data were converted into estimated distance travelled and time to travel using the R package gmapsdistance [14]. This package reports Google Maps estimates of distance travelled and time travelled between two locations. Google Maps does not provide estimates retroactively. Therefore, dates were forwarded to the corresponding day and month in the year 2024. Forwarding dates can lead to mismatching the day of the week, which can be associated with different driving conditions (e.g., weekday versus weekend). Thus, dates were manipulated so that 2024 forwarded dates matched the day of the week from the original date (e.g., 1 May 2020 (Friday) was forwarded to 1st May 2024 (Wednesday), and then corrected to 3 May 2024 (Friday)). The estimated distance and time travelled were doubled to account for travel to and from the health care facility. The estimated distance travelled and time to travel for in-person visits (excluding Manitoba Telehealth visits) were based on the postal codes of the patient’s residence and the postal code of the health care facility where the health care provider was located. The estimated distance travelled and time to travel for Manitoba Telehealth visits were based on the postal code of the patient’s residence and the postal code of the Manitoba Telehealth site used. Estimated distance travelled and time to travel for virtual visits were based on the postal code of the patient’s residence and the postal code of the health care facility where the health care provider was located.

### 2.2. Analysis

Counts by visit type (in-person and virtual) were determined for the entire study period. This was also done by age group at the time of visit (less than 18, 18 to 39, 40 to 64, 65 to 79, 80 and older), gender (men, women, other), last cancer diagnosis prior to the visit (breast, digestive, gynecologic, hematology, in situ, men genitourinary, respiratory, and other), and RHA of residence. The number of Manitoba Telehealth visits by month and the percentage of monthly visits that were virtual were reported. This was also done by age group, gender, last cancer diagnosis prior to the visit, and regional health authority of residence. The total estimated distance travelled and time travelled by month by automobile were determined. Median and 95th percentile values for estimated distance travelled and travel time for individual visits (in-person and virtual combined) were reported by area of residence. The estimated travel distance was converted into estimated metric tons of CO_2_ emissions: 206 g of CO_2_ per kilometer, which is an average based on newly registered vehicles in 2017 in Canada [15]. Google Maps did not find an estimated travel distance and travel time for 0.6% of visits. Therefore, values were imputed by using the median value of the corresponding area of residence. R version 4.1.3 was used for analyses and included the R packages of ggplot2 and gmapsdistance [14,16].

## 3. Results

### 3.1. Visits

More than 306,000 visits occurred for invasive and in situ cancers including benign brain and central nervous system tumours from April 2020 to December 2022 (Table 1). Less than half of the visits occurred virtually. More than half of the visits were with male individuals. More than half of visits occurred for individuals residing in Winnipeg, which is the largest city in the province. Approximately half the visits occurred for patients with either breast, digestive, men’s genitourinary, or hematological cancers.

### 3.2. Overall Trends

**Figure 1:**
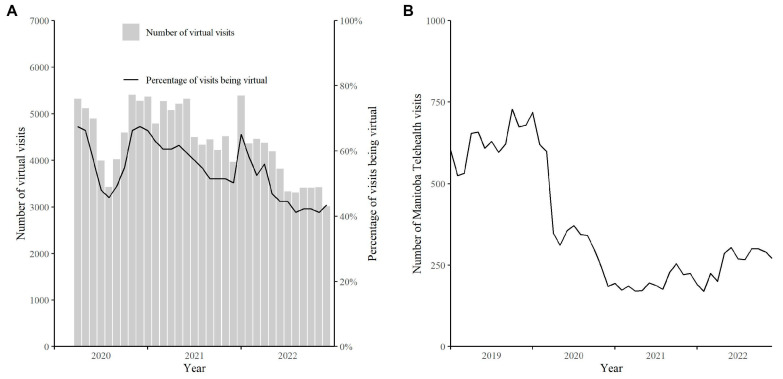
(**A**) Percentage of virtual visits by month from 1 April 2020 to 31 December 2022 in Manitoba, Canada and (**B**) number of Manitoba Telehealth visits by month from 1 April 2020 to 31 December 2022 in Manitoba, Canada.

The percentage of visits by month that occurred virtually is shown in Figure 1A. The months with the highest rate of virtual visits occurred during months associated with COVID-19 lockdowns (April and May 2020, November 2020 to January 2021, and January to February 2022) [17]. A rapid drop in virtual visits occurred after the first lockdown, followed by a rapid increase during the fall of 2020 and winter of 2020/21. The rate of virtual visits then demonstrated a slow but sustained decrease until the third lockdown, when the peak rate of virtual visits reached similar percentages as during the first and second lockdowns. The rate of virtual visits decreased steadily during 2022. An increase in Telehealth visits was observed in 2019. However, the number of Telehealth visits decreased substantially during April 2020 when the first lockdown occurred (Figure 1B). Although counts were still substantially lower than pre-pandemic levels, Telehealth visits demonstrated an increase from mid-2021 to the end of 2022.

### 3.3. Subgroup Trends

**Figure 2:**
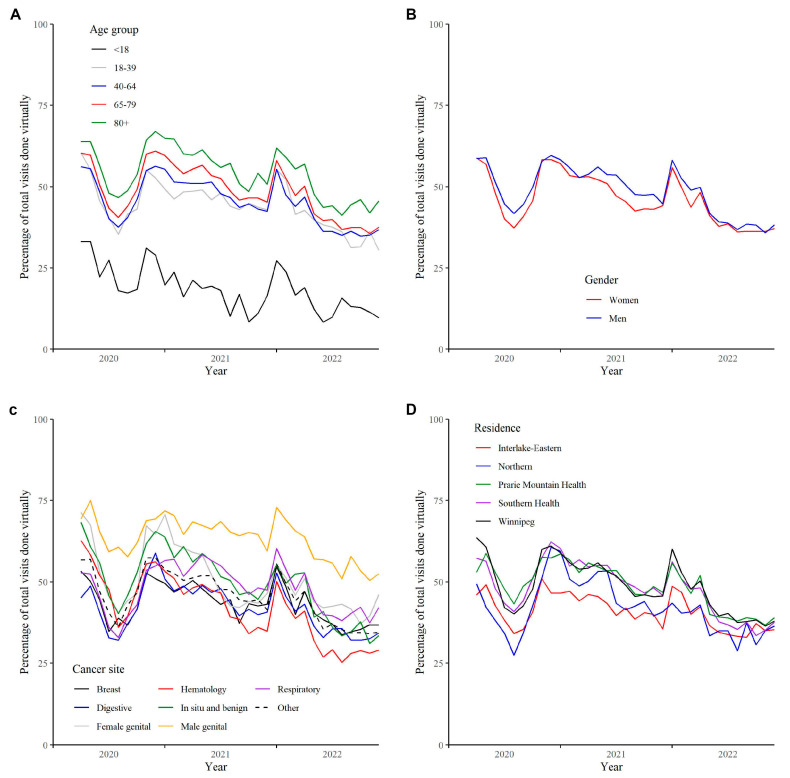
Percentage of virtual visits by month from 1 April 2020 to 31 December 2022 in Manitoba, Canada by (**A**) age group, (**B**) gender, (**C**) cancer site, and (**D**) area of residence.

The percentage of visits by month done virtually by age group is shown in Figure 2A. The percentage of virtual visits increased with age. The largest increase in percentage was between those 18 years of age and younger compared to those 18 to 39 years of age. The percentage of visits by month done virtually by gender is shown in Figure 2B. Men generally demonstrated a higher rate of virtual visits during the study period. However, smaller differences were found during months of COVID-19 lockdowns in Manitoba. The percentage of visits by month done virtually by cancer site is shown in Figure 2C. Wide variability was found between cancer sites. In addition to April 2020, men’s genitourinary cancers were consistently the site with the highest rate of virtual visits. Digestive cancers had the lowest rate of virtual visits for the year 2020, whereas hematological cancers had the lowest rates for parts of 2021 and all of 2022. Table 2 includes the percentage of visits done virtually by gender for cancer sites excluding women and men with genitourinary cancers and breast cancer. Results indicate similar rates of virtual visits between genders for hematological, as well as in situ cancers and benign brain and central nervous system tumours. However, slightly higher rates of virtual visits were found for women with digestive cancers, and higher rates for respiratory and other cancers.

The median and 95th percentile of estimated travel distance and travel time for individual visits (in person and virtual combined) by RHA of residence are presented in Table 3. Winnipeg demonstrates the shortest travel distance and time, whereas residents in Northern Manitoba demonstrate the longest travel distance and time. The percentage of virtual visits by month by area of residence is shown in Figure 2D. The Northern region (which is the region located furthest away from CCMB’s primary cancer centre located in Winnipeg) often had the lowest rates of virtual visits during 2020 and 2022, but increased to among the highest rates during part of 2021. The Interlake–Eastern RHA, which is located in the central area of the province, was consistently among the regions with the lowest monthly rates of virtual visits throughout the study period. 

### 3.4. Population Impact

**Figure 3:**
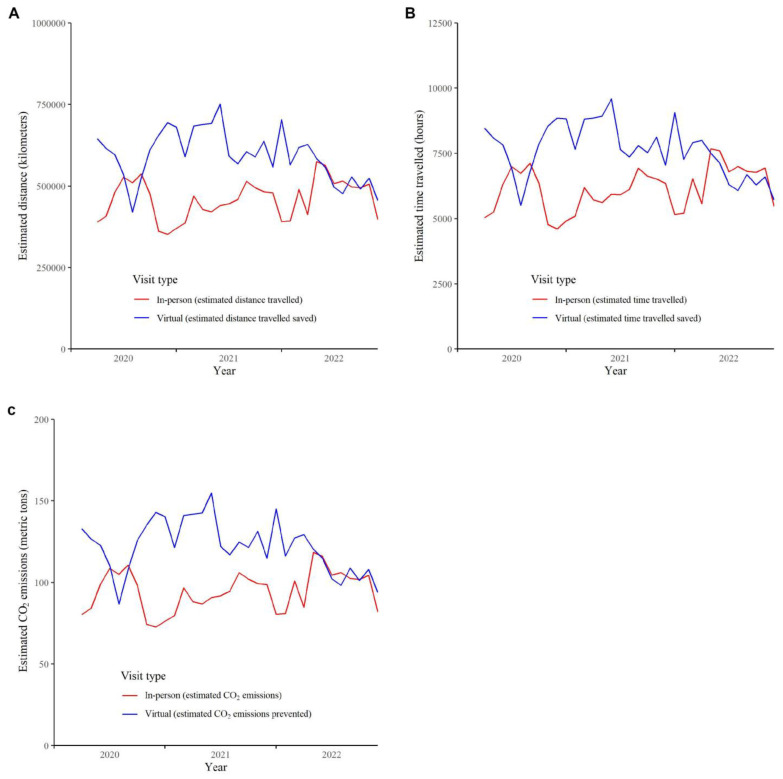
Estimated (**A**) travel distance in kilometers, (**B**) travel distance in hours, and (**C**) CO_2_ emissions prevented by month from 1 April 2020 to 31 December 2022 in Manitoba, Canada.

The total estimated travel distance saved during the study period varied from 420,000 to 750,000 km per month (Figure 3A). The total estimated travel time saved varied from 5500 to 9600 h of driving per month (Figure 3B). The estimated CO_2_ emissions prevented during the study period varied from 87 to 155 metric tons per month (Figure 3C).

## 4. Discussion

This study demonstrates the quick adoption of virtual care for cancer during the COVID-19 pandemic when individuals diagnosed with cancer could, from their residences, use both telephone calls and Microsoft Teams to interact with CCMB health care providers. As the COVID-19 pandemic progressed, we found a decreasing trend of using virtual visits over time. However, rates of virtual visits at the end of 2022 were similar to the lowest rates found between the first and second COVID-19 lockdowns, which may represent typical rates of use in the future. More data are required to verify this trend. Although telemedicine (i.e., Manitoba Telehealth) was present prior to the pandemic, its use demonstrated a substantial and immediate decrease, which was likely due to its replacement by virtual visits from the patient’s residence because of the restrictions placed on visiting a health care facility. Overall though, the findings from this study demonstrate that virtual visits have been widely utilized to deliver cancer care throughout the population, saving individuals travel time and resulting in diminished CO_2_ emissions.

We found that older individuals, men, and individuals diagnosed with male genitourinary cancers were more likely to participate in virtual visits. During the study time period, family members were not allowed to accompany adult patients to clinic visits to reduce person-to-person contact. For elderly patients, the use of virtual visits likely allowed patients to receive care without needing to leave their place of residence, permitted family members to participate in clinic visits with older relatives (sometimes through conference calls), and eliminated the need for adult children to leave work to accompany an elderly parent to in-person visits. In contrast, for patients under 18, the relatively low use of virtual care suggests that patients, clinicians, and parents/guardians had less comfort with virtual care. Moreover, during the pandemic, patients under 18 were allowed to have a parent or guardian accompany them making the use of virtual visits less necessary. However, further research is needed in the pediatric population, because in addition to the reduction of environmental impact, based on time saved from travel alone, there may be additional important benefits of virtual care for families participating in the care of a pediatric patient.

In terms of gender differences between use of virtual visits, virtual care use was highest for men’s genitourinary cancers likely because many of these individuals were on active surveillance for prostate cancer and an in-person visit was not needed. However, when rates for cancer sites were stratified by sex, women generally demonstrated similar or sometimes higher rates of virtual visit use suggesting that gender, in and of itself, likely does not play a role in virtual care usage. Lastly, although the Northern region of the province is very large and remote, requiring significant travel for most residents, patients in the north often had the lowest rate of virtual visits. This could potentially be due to personal preference, the need for an extended stay in a larger centre for multiple encounters, or technology limitations. However, given the resources involved in transporting individuals from northern communities, identifying barriers to virtual care for this population is important in terms of both optimizing health care resources and the patient experience. Future work should also examine differences in virtual visit use by other social determinants of health such as income level and time since immigration.

Virtual visits have the potential to yield significant environmental benefits. Virtual visits clearly reduced the resources required for care as they saved both travel distance and reduce CO_2_ emissions with an estimated 420,000 to 750,000 km in travel distance and 87 to 155 metric tons of CO_2_ emissions saved per month during the study period. Several other studies have also estimated substantial reductions of CO_2_ emissions with the use of virtual visits in health care [18,19,20]. Another important benefit reported in the literature through the use of virtual visits is decreased costs due to reduced travelling [18,20,21,22]. Our findings reflect another important benefit reported in the literature through the use of virtual visits, namely, decreased costs to patients and their families resulting from reduced travelling [18,20,21,22]. As we identified in this study, even individuals who live near major cancer centres lose travel time to traffic, parking, and weather, all of which can be avoided with virtual visits. Lower production losses (e.g., absence from work) for both patients and informal caregivers have also been reported [5,20,23]. Virtual visits also allow more informal caregivers to be present [5]. However, virtual visits may not be appropriate for all types of visits such as initial visits, visits that require a physical examination, or those that include potentially difficult or sensitive conversations [5]. Although studies investigating the impact of virtual visits have generally reported high satisfaction by patients in a variety of settings [19,21,23], variable reactions have been reported within a sample of individuals diagnosed with cancer [5]. In the future, guidelines regarding the optimal use of virtual visits are needed, as well as conversations between health care providers and patients so that these decisions can be made jointly, taking into consideration patient preferences. Due to the benefits provided through virtual visits, the variability of its use by subgroups, and the potential decreasing trend of its use over time, efforts might be needed to encourage virtual visits for interactions where in-person visits are not essential. It is also important to consider the requirements of funding and regulatory bodies in examining the feasibility of continuing virtual medicine use beyond the pandemic situation.

Some limitations of the current study include assuming that everyone travelled by automobile. Some remote regions are not accessible by automobile, which would have required different modes of transportation such as flying in an airplane. Public transportation could also have been used in larger urban areas, which could underestimate travel time, although not impact the estimated decrease in CO_2_ emissions since public transport occurs regardless of whether or not there is virtual care. Some patients could have had an extended stay in a city for multiple visits, therefore leading to overestimates in travel time and distance. Median imputation by area of residence assumes that missing data are similar to non-missing data. However, missing data were minimal. The average CO_2_ emissions from 2017 vehicles do not include newer vehicles which would have lower values, but also do not include older vehicles which may have higher values. More accurate data could be obtained about vehicle details and public transportation use by surveying cancer patients. This would improve the accuracy of the estimates but would also require a high response rate and would be costly. Assuming that the last cancer diagnosed is associated with the current visit could lead to misclassification of cancer type if an individual had multiple primaries (e.g., distinguishing between cancer recurrence, progression, and new primary).

## 5. Conclusions

Virtual visits were introduced almost immediately at the start of the COVID-19 pandemic when more than half of visits were conducted virtually. The use of virtual visits differed by age group, sex, cancer type, and area of residence. Substantial estimated travel distance, travel time, and CO_2_ emissions have been prevented through the introduction of virtual visits. The identification and elimination of barriers to virtual visits, as well as the development of evidence-based guidelines for appropriate virtual visit use, are needed for the optimal incorporation of virtual care into usual cancer care. We posit that virtual care is an important tool for better supporting those living with cancer, and for providing a meaningful contribution to the critical need to decrease greenhouse gas emissions.

## Figures and Tables

**Table 1:** Description of visits to CanerCare Manitoba from 1 April 2020 to 31 December 2022.

		*N*	%
Total		306,234	
Type	In person	160,668	52.5
	Virtual	145,566	47.5
Age group	Under 18	5198	1.7
	18–39	13,805	4.5
	40–4	108,457	35.4
	65–79	137,951	45.0
	80 and older	40,823	13.3
Gender	Women	147,178	48.1
	Men	159,043	51.9
	Other	13	0.0
Regional Health Authority of residence	Interlake–Eastern	34,403	11.2
	Northern	9756	3.2
	Prairie Mountain Health	46,706	15.3
	Southern Health	44,882	14.7
	Winnipeg	170,487	55.7
Cancer site	Breast	41,178	13.4
	Digestive	45,957	15.0
	Gynecologic	21,514	7.0
	Hematology	45,879	15.0
	In situ and benign	23,774	7.8
	Men’s genitourinary	42,571	13.9
	Respiratory	30,093	9.8
	Other	55,268	18.0
Missing travel data		1729	0.6

**Table 2:** Percentage of visits done virtually by cancer site, year of visit, and gender.

	2020		2021		2022	
Cancer Site	Women	Men	Women	Men	Women	Men
Digestive	44.0	42.5	47.1	44.0	40.3	36.3
Hematology	51.8	49.6	44.8	42.9	34.2	33.1
In situ and benign *	54.8	55.4	52.3	54.0	40.5	40.9
Respiratory	47.9	42.8	55.0	50.1	48.2	41.2
Other	52.7	46.5	51.2	46.6	42.9	38.3

* Includes benign brain and central nervous system cancers.

**Table 3:** Median and 95th percentile of estimated distance (km) and travel time (minutes) for individual visits by Regional Health Authority of residence (in person and virtual combined).

	Travel Distance (km)		Travel Time (min)	
Regional Health Authority of Residence	Median	95th Percentile	Median	95th Percentile
Interlake–Eastern	106	370	86	253
Northern	1244	1623	764	1044
Prairie Mountain Health	162	708	111	470
Southern Health	119	290	94	214
Winnipeg	18	33	30	52

## Data Availability

The data that support the findings of this study are not publicly available to ensure and maintain the privacy and confidentiality of individuals’ health information. Requests for data may be made to the appropriate data stewards (CancerCare Manitoba’s Research and Resource Impact Committee).

## Supplementary Materials

The following supporting information can be downloaded at: https://www.mdpi.com/article/10.3390/curroncol30070446/s1, Figure S1: Map of Manitoba’s Regional Health Authorities.
