# Supplementary material for: Impact of Cancer-Related Virtual Visits on Travel Distance, Travel Time, and Carbon Dioxide (CO2) Emissions during the COVID-19 Pandemic in Manitoba, Canada
Source: Curr Oncol. 2023 Jun 21;30(7):5973–83. doi: 10.3390/curroncol30070446 (PMC10378106; doi:10.3390/curroncol30070446)
Supplement: Supplementary file 1 [file curroncol-30-00446-s001.zip › curroncol-2380864-supplementary.pdf]

Supplemental Figures

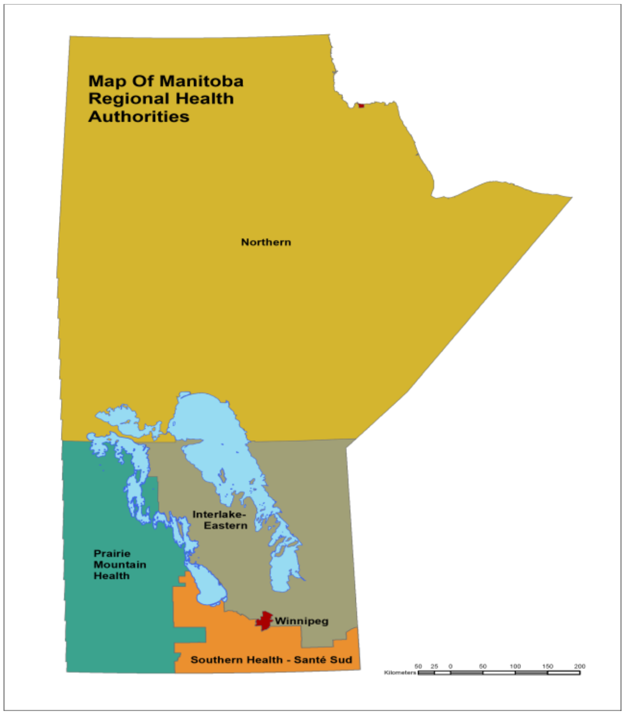

Figure S1. Map of Manitoba's Regional Health Authorities

Source: https://www.gov.mb.ca/health/rha/docs/map.pdf
